# Impact of postoperative necrotizing enterocolitis after neonatal cardiac surgery on neurodevelopmental outcome at 1 year of age

**DOI:** 10.3389/fped.2024.1380582

**Published:** 2024-08-06

**Authors:** Walter Knirsch, Alexandra De Silvestro, Verena Rathke, Christelle L’Ebraly, Julia C. Natterer, Juliane Schneider, Nicole Sekarski, Beatrice Latal, Cristina Borradori-Tolsa, Maya S. Bouhabib, Katharina Fuhrer Kradolfer, Martin Glöckler, Damian Hutter, Marc R. Pfluger, Lena Kaiser, Angelo Polito, Janet F. Kelly-Geyer, Michael von Rhein, the Swiss ORCHID group, Nicole Sekarski

**Affiliations:** ^1^ Pediatric Cardiology, Pediatric Heart Center, Children's Research Center, University Children's Hospital, University of Zurich, Zurich, Switzerland; ^2^ Pediatric Cardiology, Woman-Mother-Child Department, University Hospital Lausanne, Lausanne, Switzerland; ^3^ Pediatric Cardiology, Woman-Child-Adolescent Department, University Hospitals and Faculty of Medicine, University of Geneva, Geneva, Switzerland; ^4^ Pediatric Intensive Care Unit, Woman-Mother-Child Department, University Hospital Lausanne, Lausanne, Switzerland; ^5^ Neonatology, Woman-Mother-Child Department, University Hospital Lausanne, Lausanne, Switzerland; ^6^ Child Development Center, Children's Research Center, University Children's Hospital, University of Zurich, Zurich, Switzerland; ^7^ Development and Growth, Department of Pediatrics, University Hospitals and Faculty of Medicine, University of Geneva, Geneva, Switzerland; ^8^ Pediatric Neurology, Bern, Switzerland; ^9^ Pediatric Cardiology, Center for Congenital Heart Disease, Department of Cardiology and Cardiac Surgery, University Children's Hospital, University of Bern, Bern, Switzerland; ^10^ Pediatric and Neonatal Intensive Care Unit, Department of Pediatrics, Gynecology and Obstetrics, University Hospitals and Faculty of Medicine, University of Geneva, Geneva, Switzerland; ^11^ Department of Neonatology and Pediatric Intensive Care, Children's Research Center, University Children's Hospital, University of Zurich, Zurich, Switzerland; ^12^ Swiss Neurodevelopmental Outcome Registry for Children with CHD ORCHID, Switzerland

**Keywords:** congenital heart disease, neurodevelopmental outcome, neonates, cardiopulmonary bypass surgery, complications

## Abstract

**Objectives:**

We analyzed the impact of postoperative necrotizing enterocolitis (NEC) after cardiac surgery in neonatal age on neurodevelopmental (ND) outcome at 1 year of age.

**Methods:**

Using data from the Swiss Neurodevelopmental Outcome Registry for Children with Congenital Heart Disease (ORCHID), we analyzed perioperative variables including postoperative NEC (Bell's stage ≥2) and 1-year ND outcome (Bayley III).

**Results:**

The included patients (*n* = 101) had congenital heart disease (CHD), categorized as follows: 77 underwent biventricular repair for CHD with two functional chambers, 22 underwent staged palliation until the Fontan procedure for CHD with single ventricle physiology (*n* = 22), or 4 underwent single ventricle palliation or biventricular repair for borderline CHD (*n* = 4). Neonatal cardiopulmonary bypass (CBP) surgery was performed at a median age (IQR) of 8 (6) days. NEC occurred in 16 patients. Intensive care unit (ICU) length of stay (LOS) and the total duration of the hospitalization were longer in children with NEC than those in others (14 with vs. 8 days without NEC, *p* < 0.05; 49 with vs. 32 days without NEC, *p* < 0.05). The Bayley III scores of the analyzed patients determined at an age of 11.5 ± 1.5 months showed cognitive (CCS) (102.2 ± 15.0) and language scores (LCS) (93.8 ± 13.1) in the normal range and motor composite scores (MCS) (88.7 ± 15.9) in the low-normal range. After adjusting for socioeconomic status and CHD type, patients with NEC had lower CCS scores [*β* = −11.2 (SE 5.6), *p* = 0.049]. Using a cumulative risk score including NEC, we found a higher risk score to be associated with both lower CCS [*β* = −2.8 (SE 1.3), *p* = 0.030] and lower MCS [*β* = −3.20 (SE 1.3), *p* = 0.016].

**Conclusions:**

Postoperative NEC is associated with longer ICU and hospital LOS and contributes together with other complications to impaired ND outcome at 1 year of age. In the future, national and international patient registries may provide the opportunity to analyze large cohorts and better identify the impact of modifiable perioperative risk factors on ND outcome.

**Clinical Trial Registration:**

ClinicalTrials.gov identifier: NCT05996211.

## Introduction

Necrotizing enterocolitis (NEC) is a severe postnatal complication most commonly seen in preterm and low birth weight newborns and is associated with a high risk of morbidity and mortality (1). NEC is an acute inflammatory disease usually affecting parts of the small intestine. It presents with clinical findings including bloody stool, abdominal distension, and sepsis and may deteriorate with bowel necrosis and bowel perforation resulting in devasting disease (2). The etiology of NEC is multifactorial and not completely understood (2, 3). Risk factors for NEC include prematurity, low birth weight, formula feeding, hypoxic/ischemic insults, and microbial dysbiosis (1). Populations at risk for NEC include neonates with severe types of congenital heart disease (CHD) and then considered *cardiogenic NEC*, which has been differentiated from classical NEC of preterm newborns (4). In terms of localization, cardiogenic NEC tends to be mainly present in the colon, and NEC in preterm newborns tends to mainly involve the small intestine. In neonates with cardiogenic NEC, most frequently due to a complex type of CHD, either an unbalanced ratio of pulmonary (Qp) to systemic (Qs) perfusion or perioperative low cardiac output syndrome can lead to mesenteric hypoperfusion with hypoxic and/or ischemic results and the clinical start of NEC (5).

Besides this mesenteric hypoperfusion, the low systemic cardiac output (Qs) may also lead to cerebral hypoperfusion. Cerebral hypoxia/ischemia by itself, independent of NEC, has been described as a potential risk factor for altered brain development before and after birth (6, 7), including altered brain growth and postnatal structural alterations, of which white matter lesions and liquor space enlargement play a crucial role for impaired long-term neurodevelopmental (ND) outcome (8).

The link between NEC and impaired ND outcome may either be due to comparable hemodynamics of low cerebral and low mesenteric blood perfusion with its described consequences and/or secondary to clinical consequences of NEC such as impaired enteral diet and caloric intake and prolonged intensive care unit (ICU) and hospital length of stay. Therefore, we aimed to discriminate the impact of NEC as an additional risk factor for neurodevelopmental disorders in infants with congenital heart disease, using data from the Swiss Neurodevelopmental Outcome Registry for Children with Complex Congenital Heart Disease (ORCHID) registry (9).

## Patients and methods

### Data source

The analysis is based on data from the Swiss Neurodevelopmental Outcome Registry for Children with Complex Congenital Heart Disease (ORCHID), a nationwide registry (founded in 2018) on the ND outcome of patients undergoing early neonatal cardiac surgery or hybrid palliation at <6 weeks of age (9).

### Patients

We included all consecutively registered patients of the ORCHID registry born and treated between 2019 and 2021. We grouped into those with or without the diagnosis of postoperative cardiogenic NEC according to Bell's stage ≥2, including infants with abdominal distension, blood in stool combined with bowel edema, pneumatosis, pneumoperitoneum determined by abdominal radiograph (x-ray), or abdominal ultrasound (10).

### Clinical variables

We collected and analyzed baseline characteristics, patient information, presurgical data such as type and classification of CHD, anthropometric data, sex, and periprocedural medical, surgical, and intensive care data for the first main surgical or catheter-based stage I procedure. We also documented the need for reinterventions including surgery and catheter-based procedures, complications, and clinical and ND outcome at 1 year of age [for details see also (9)].

A risk score was calculated for each patient as a cumulative score including NEC occurrence, complications as defined as “other complications” in Table 1, and need for ECMO and resuscitation. Socioeconomic status (SES) was defined as the mother's highest education level plus the father's current occupation (11).

**Table 1:** Comparison of patients with and without NEC undergoing neonatal cardiac surgery.

	Patients, all (*n* = 101)	Patients without NEC (*n* = 85)	Patients with NEC (*n* = 16)	*P*-value
Type of CHD				0.658
Single ventricle CHD	22 (22%)	18 (21%)	4 (25%)	* *
Biventricular CHD	75 (74%)	64 (75%)	11 (69%)	* *
Borderline CHD	4 (4%)	3 (4%)	1 (6%)	* *
Type of surgery (RACHS-1) (*n*=)				0.472
I	0	0	0	* *
II	10 (10%)	8 (9%)	2 (13%)	* *
III	40 (40%)	36 (42%)	4 (25%)	* *
IV	35 (35%)	29 (34%)	6 (38%)	* *
V	2 (2%)	2 (2%)	0	* *
VI	14 (14%)	10 (12%)	4 (25%)	* *
CPB, yes	87 (86%)	72 (85%)	15 (94%)	0.459
CPB time (min)	203 (161–270)	207 (157–270)	197 (180–270)	0.968
Cross-clamping time (min)	128 (95–158)	129 (85–158)	120 (115–163)	0.738
ECMO (*n*=)	9 (9%)	6 (7%)	3 (19%)	0.151
Resuscitation (*n*=)	11 (11%)	11 (13%)	0	0.205
ICU LOS (days)	9 (6–22)	8 (6–21)	14 (10–42)	**0.045**
LOHS (days)	34 (22–52)	32 (22–50)	49 (35–79)	**0.041**
Complications (*n*=)				
Infection/sepsis	15 (15%)	12 (14%)	3 (19%)	0.702
Wound infection	11 (11%)	8 (9%)	3 (19%)	0.374
Diaphragmatic paralysis	4 (4%)	4 (5%)	0	1.000
Chylothorax	15 (15%)	11 (13%)	4 (25%)	0.250
Respiratory complications	35 (35%)	27 (32%)	8 (50%)	0.251
Cerebral stroke	1 (1%)	1 (1%)	0	1.000
Cerebral seizures	1 (1%)	0	1 (6%)	0.158
Cerebral MRI, pathologic	14 (14%)	12 (14%)	2 (13%)	1.000
Total number of complications (*n*=)	1 (0–1)	1 (0–1)	1 (0–2)	0.156
Risk score of complications	1 (0–2)	1 (0–2)	2 (1–3)	**<0**.**001**
Socioeconomic status	4 (3–6)	5 (3–6)	4 (4–5)	0.344
ND Outcome at 1 year (Bayley III)				
CCS	102.2 ± 15.0	103.5 ± 14.1	95.0 ± 15.8	0.070
LCS	93.8 ± 13.1	94.4 ± 13.1	90.6 ± 13.4	0.359
MCS	88.7 ± 15.9	89.0 ± 15.7	87.0 ± 17.6	0.685
Somatic growth at 1 year				
Body length (*z*-score)	0.02 ± 1.30	0.04 ± 1.26	−0.14 ± 1.55	0.632
Body weight (*z*-score)	−0.19 ± 1.20	−0.15 ± 1.16	−0.38 ± 1.40	0.499
Head circumference (*z*-score)	−0.46 ± 1.53	−0.35 ± 1.46	−1.05 ± 1.81	0.106

Data are reported as median (IQR) and mean ± SD, as appropriate. Socioeconomic status was missing in 10 patients (6 without NEC, 4 with NEC). Bayley Scales were missing in 22 patients (18 without NEC, 4 with NEC).

Bold *P* values are significant (*P* < 0.05).

Bayley III, Bayley Scales of Infant and Toddler Development, Third Edition; CCS, cognitive composite score; CHD, congenital heart disease; CPB, cardiopulmonary bypass; ECMO, extracorporeal membrane oxygenation; ICU LOS, intensive care unit length of stay; LCS, language composite score; LOHS, length of hospital stay; MCS, motor composite score; ND, neurodevelopmental; RACHS-1, risk adjustment for congenital heart surgery-1.

### Neurodevelopmental outcome

To assess cognitive (receptive and expressive) language and (fine and gross) motor development at 1 year of age, we used the Bayley Scales of Infant and Toddler Development, Third Edition (Bayley III), with the respective composite scores [cognitive composite score (CCS), language composite score (LCS), motor composite score (MCS)] (12).

### Statistics

Statistical analyses were conducted using R (version 4.1.2, The R Software Foundation for Statistical Computing, Vienna, Austria). Univariate analysis between patients with and without NEC was conducted using an unpaired *t*-test for normally distributed data, Mann–Whitney *U*-test for non-normally distributed data, and Fisher's exact test for categorical data. The associations of NEC occurrence and risk score with ND outcome were further evaluated by applying a multivariate linear regression model, adjusting for SES and CHD categories (univentricular vs. biventricular or borderline).

### Ethics

The Swiss ORCHID registry has been reviewed by the cantonal ethics committees (Req-2019-00089), and for the study analysis of the impact of NEC on ND outcome (BASEC-2022-00689).

## Results

Between 2019 and 2021, we included 101 patients (63 female) with different types of CHD undergoing neonatal cardiac surgery within the first 6 weeks of life in the ORCHID registry. Cardiac diagnoses were CHD undergoing definitive cardiac biventricular repair (*n* = 75), such as D-transposition of the great arteries (TGA) with arterial switch operation or palliative stage I procedure in neonates with single ventricle CHD (*n* = 22), such as hypoplastic left heart syndrome (HLHS) with Norwood I procedure. In addition, patients with a borderline type of CHD (*n* = 4) were included. Most of the patients underwent neonatal cardiopulmonary bypass (CPB) surgery (*n* = 86), at a median (IQR) age of 8 (6) days, and the severity of the first surgical procedure was moderate as determined by the Risk Adjustment for Congenital Heart Surgery-1 (RACHS-1) scores 3–4 in 75% of the patients (Table 1). Median CPB time was 203 (IQR 161–270) min with a median (IQR) cross-clamping time of 128 (95–158) min at a moderate core temperature of 31.2°C ± 4.0°C. More than half of the patients (*n* = 53) underwent CPB surgery with selective cerebral perfusion.

After surgery, NEC occurred in 16 patients, which was the second most frequent complication after respiratory complications (*n* = 35) of the whole cohort (Table 1). Clinical manifestation of NEC, findings of abdominal x-ray, and abdominal ultrasound resulted in Bell’s stage II A (*n* = 9), stage II B (*n* = 6), and stage III B (*n* = 1), respectively (Table 2). NEC occurred after neonatal cardiac surgery at a median of 6.5 days postoperatively (IQR 4–14.75), at a postnatal age of 22 (11.5–30.75) days. Half of the patients with postoperative NEC were treated for HLHS (stage I Norwood procedure) or TGA (arterial switch). The type of CHD, the severity of cardiac surgery determined by RACHS-1 score, and the use of cardiopulmonary bypass were not associated with a higher risk for NEC. The use of postoperative ECMO or resuscitation was comparable in patients with or without NEC (Table 1). NEC was treated by intravenous antibiotics and parental nutrition for at least 5 days (*n* = 16), while one infant needed abdominal surgery (Table 2). This infant was treated for obstructive total anomalous pulmonary vein drainage by sutureless repair with the need for a secondary redo after 2 days. Despite intravenous antibiotics and parental nutrition, surgery became necessary including colon resection plus colostomy (Table 2).

**Table 2:** Diagnosis, time, and CHD type of patients with complicating NEC after neonatal cardiac surgery.

Pat	Clinical manifestation	x-ray	US	Bell's stage	Age at NEC (days)	NEC postsurgery (days)	CHD type	Type of surgery	Cofactors	Treatment of NEC	Outcome
# 1	+ emesis, no blood	+	++	IIb	118	28	SV (TA IB)	Shunt procedure (mBTS re), PAB	Preterm	Parenteral feeding/antibiotics (two times for 5 days)	Alive
# 2	++ hemotochezia, abdominal tenderness	+	+	IIa	6	5	SV (HLHS)	Norwood I (mBTS)	Preterm	Parenteral feeding/antibiotics (for 5 days)	Alive
# 3	++ hematochezia, low bowel sounds	++	++	IIb	19	6	Complex TGA	ASO complex	ECMO	Parenteral feeding/antibiotics (for 5 days)	Alive
# 4	−	+++	++	IIb	4	1	Simple TGA	ASO	BAS, neonatal infection	Parenteral feeding/antibiotics (for 5 days)	Alive
# 5	++ hematochezia, abdominal tenderness	++	+	IIa	30	23	SV (HLHS)	Norwood I (mBTS)	−	Parenteral feeding/antibiotics (for 5 days)	Alive
# 6	+ hematochezia	++	+	IIa	23	17	Complex TGA	ASO complex	−	Parenteral feeding/antibiotics (for 5 days)	Alive
# 7	+++ hematochezia, low bowel sounds, abdominal tenderness	++	+++	IIIb	34	22	TAPVD	Sutureless repair	−	Parenteral feeding/antibiotics (for 10 days)/surgery	Alive
# 8	−	++	+	IIa	13	7	SV (HLHS)	Norwood I (mBTS)	−	Parenteral feeding/antibiotics (for 5 days)	Alive
# 9	++ hematochezia, low abdominal tenderness	++	++	IIb	4	1	Truncus arteriosus	Pulmonary artery banding	−	Parenteral feeding/antibiotics (for 5 days)	Alive
# 10	+ no blood, but abdominal tenderness	+	++	IIb	26	4	VSD, ASD	VSD/ASD Closure	−	Parenteral feeding/antibiotics (for 5 days)	Alive
# 11	++ hematochezia, abdominal tenderness	+	+	IIa	55	14	Aortic atresia	PAB bil, Yasui + redo	ECMO	Parenteral feeding/antibiotics (for 5 days)	Alive
# 12	+ emesis, no blood, abdominal tenderness	+	+	IIa	22	7	Aortic valve stenosis	Aortic valve surgery	−	Parenteral feeding/antibiotics (for 5 days)	Alive
# 13	+ no blood, but abdominal tenderness	+	++	IIb	22	8	Truncus arteriosus	RV-PA Sano Shunt	22q11	Parenteral feeding/antibiotics (for 5 days)	Alive
# 14	+ hematochezia persistent	++	+	IIa	33	1	TAPVD, obstructive	Sutureless repair plus redo	ECMO	Parenteral feeding/antibiotics (for 10 days)	Alive
# 15	+ emesis, no blood	++	++	IIa	12	5	Hemitruncus	RPA reimplantation	−	Parenteral feeding/antibiotics (for 5 days)	Alive
# 16	++ emesis, hematochezia	+	++	IIa	10	4	SV (HLHS)	Norwood stage I	−	Parenteral feeding/antibiotics (for 5 days)	Alive

ASD, atrial septal defect; ASO, arterial switch operation; BAS, balloon atrioseptostomy; CHD, congenital heart disease; ECMO, extracorporeal membrane oxygenation; HLHS, hypoplastic left heart syndrome; mBTS, modified Blalock–Taussig shunt; NEC, necrotizing enterocolitis; PAB bil., bilateral pulmonary artery banding; RPA, right pulmonary artery; SV, single ventricle; TA, tricuspid atresia; TAPVD, total abnormal pulmonary vein drainage; TGA, transposition of great arteries; US, ultrasound; VSD, ventricular septal defect; 22q11, microdeletion syndrome 22q11.

The severity of clinical manifestation, x-ray, and US was labeled as no −, mild +, moderate ++, and severe +++.

Infants with postoperative NEC had a longer ICU and total hospital stay (Table 1). Postoperative NEC was associated with increased postoperative ICU stay by more than 50% [postoperative ICU time with complicating NEC median (IQR) 14 (10–42) days vs. no NEC 8 (6–21) days, *p* = 0.045] and increased total hospital stay by more than 2 weeks [total hospital stay with complicating NEC 49 (35–79) days vs. no NEC 32 (22–50) days, *p* = 0.041] (Table 1). The overall number of other complications was not higher in the NEC population as well as comparing the patient individual number of complications between postoperative NEC and no NEC (Table 1).

The ND outcome was determined by the Bayley III at a mean (±SD) age of 11.5 ± 1.5 months. For the whole patient population, the CCS was mean (SD) 102.2 (15.0), the LCS was 93.8 (13.1), and the MCS was 88.7 (15.9).

All three scores were lower in the NEC population, but without evidence of significance (Table 1). Clinical outcome data are presented in Table 2. When adjusting the analysis for SES and CHD type (Table 3), patients with NEC had lower CCS scores than those in patients without [*β* = −11.2 (SE 5.6), *p* = 0.049]. However, results in LCS and MCS remained similar. Multiple *R*^2^ was 0.08 for the CCS, 0.08 for the MCS, and 0.07 for the LCS model.

**Table 3:** Association of NEC, CHD type, and socioeconomic status with Bayley composite scores.

	Beta estimate	Standard error	*P*-value
Cognitive composite score			
Intercept	106.21	4.61	<0.001
NEC: yes	−11.16	5.57	0.049
CHD type: univentricular	−5.28	4.51	0.25
Socioeconomic status	−0.26	0.82	0.75
Language composite score			
Intercept	95.70	4.07	<0.001
NEC: yes	−5.43	4.93	0.27
CHD type: univentricular	−6.84	3.98	0.09
Socioeconomic status	0.03	0.72	0.97
Motor composite score			
Intercept	97.96	4.83	<0.001
NEC: yes	−6.14	5.84	0.30
CHD type: univentricular	−5.02	4.73	0.29
Socioeconomic status	−1.60	0.86	0.07

Analyzing the same models using the cumulative risk score instead of the presence of NEC only (Table 4), we found a higher risk score to be associated with both lower CCS [*β* = −2.8 (SE 1.3), *p* = 0.030] and lower MCS [*β* = −3.20 (SE 1.3), *p* = 0.016], but not with lower LCS [*β* = −0.7 (SE 1.1), *p* = 0.55]. Multiple *R*^2^ was 0.09 for the CCS, 0.14 for the MCS, and 0.05 for the LCS model.

**Table 4:** Association of risk score, CHD type, and socioeconomic status with Bayley composite scores.

	Beta estimate	Standard error	*P*-value
Cognitive Composite Score			
Intercept	107.24	4.68	<0.001
Risk score	−2.81	1.27	0.030
CHD type: univentricular	−5.56	4.46	0.22
Socioeconomic status	−0.08	0.80	0.92
Language composite score			
Intercept	95.37	4.19	<0.001
Risk score	−0.69	1.13	0.55
CHD type: univentricular	−7.19	3.99	0.08
Socioeconomic status	0.14	0.72	0.85
Motor composite score			
Intercept	100.55	4.77	<0.001
Risk score	−3.20	1.29	0.016
CHD type: univentricular	−4.67	4.54	0.31
Socioeconomic status	−1.55	0.82	0.06

## Discussion

This is the first population-based analysis of the relationship between postoperative NEC and neurodevelopmental outcome in children with severe CHD having undergone cardiac surgery in the first 6 weeks of life (9). In our study, postoperative NEC was the second most frequent complication (after postoperative respiratory complication) during ICU treatment. As expected, the occurrence of NEC was associated with prolonged length of hospital stay, but we also found a significant association with a lower cognitive outcome at 1 year of age. However, a cumulative risk score summarizing various complications was associated with both cognitive and motor outcomes and could therefore better predict ND outcome than NEC alone. *Postoperative* NEC was furthermore associated with an adverse ND outcome at 1 year of age with poorer cognitive outcome when adjusted for type of CHD and SES. Therefore, preventing or early identifying and treating NEC could help to improve the long-term ND outcome.

The high *incidence* of postoperative NEC in 16% of our ORCHID cohort may be explained by the inclusion criteria focusing on a high-risk population leading to a high number (90%) of patients with a risk score in RACHS-1 of more than class 2 (Table 1) and by our diagnostic definition of NEC including more cases diagnosed by ultrasound compared to x-ray, which might have led to overdiagnosis. However, a comparable incidence of NEC in 19.5% of patients with severe CHD (RACHS-1 >2) was reported in a recent case–control study by Gong et al. (13). This contrasts with the overall incidence of NEC (Bell's stage >2) ranging between 2% and 11% for the general population of term-born infants (4, 14, 15).

The *diagnosis* of NEC was made according to the historically defined stage ≥2 based on radiography in our study, as defined by Bell et al. in the late 1980s (10, 16). Nevertheless, the findings of pneumatosis intestinalis may to some degree be examiner-dependent and have to be interpreted in the context of the clinical findings. Other imaging modalities such as ultrasound have obvious advantages regarding their bedside availability, but their diagnostic power may be overestimated due to a larger number of false-positive results (17). Nevertheless, utilizing ultrasound in suspected NEC is at least equivocal to radiography (18, 19). Recently, Doppler ultrasonography of the superior mesenteric artery has gained importance as a sensitive diagnostic (or even prognostic) tool if performed early (20). So far, modern subtyping of the different entities of NEC may provide a clearer diagnostic approach, especially for research (21).

Certainly, cases of NEC in our study reflect the broad clinical spectrum of cardiogenic NEC including different severity levels of the affected infants due to ischemic mesenteric hypoperfusion (Table 2). The diagnosis of NEC remains challenging (17). In the past, different modifications of the Bell staging system for the preterm population have been developed trying to overcome the difficulties in diagnosing NEC including gestational age, biomarkers, genetic factors, single or multi-omics approaches, and stool microbiota (17). There is a risk of *overdiagnosing* cardiogenic NEC due to temporary postoperative low cardiac output and mesenteric ischemia; some cases suspicious for NEC in our cohort with rather nonspecific clinical signs and a suggestive abdominal radiograph or sonography were early treated due to the risk of rapid clinical deterioration of NEC (17).

Regarding the *etiology* of cardiogenic NEC, patients with severe CHD are at increased risk for mesenteric hypoperfusion followed by endothelial inflammation and increased vascular permeability due to the release of cytokines, leading to intestinal epithelial barrier dysfunction, bacterial translocation, and intestinal dysbiosis. The role of intestinal dysbiosis in CHD patients has been described as a result of reduced gut perfusion and gut hypoxemia. This may lead to a reduction of healthy bacteria and an increase of proinflammatory bacteria, with increased levels of trimethylamine N-oxide affecting the liver, reduced short-chain fatty acids, and bile acids affecting myocardial contractility (22). Furthermore, in cardiogenic NEC, microcirculatory changes in CHD patients with altered hemodynamics compared to normal infants may contribute by altered cross talk of intrinsic endothelin-1 (vasoconstrictor) and nitric oxide (vasodilator), which has been shown in animal models demonstrating a maladaptive vasoconstriction to compensate for postoperative arterial hypotension and hypoxemia (23, 24). This process may be intensified after surgery due to a potential reperfusion injury, especially in higher-risk cases involving cardiopulmonary bypass surgery and hypothermia, and may be aggravated due to a more complicated postoperative course with ongoing systematic inflammation and/or low cardiac output syndrome (25). Furthermore, the use of inotropes (26), ECMO (27), and blood transfusions have been associated with an increased risk of NEC.

Before surgery, different *prevention strategies* including feeding guidelines for neonates have been developed for high- vs. low-risk patients (28). High-risk infants included HLHS, truncus arteriosus, single ventricle, and duct-dependent systemic blood flow. Other risk factors for cardiogenic NEC include concomitant premature birth (<37 weeks of gestation), low birth weight (<2,500 g), high RACHS-1 scores (>2), and trisomy 21. Evidence-based postoperative feeding strategies, however, are lacking. The risk of early postoperative NEC has been determined within the first 3 days after surgery (even before starting enteral feeding), as well as due to suboptimal caloric intake, catabolic stress, critical illness leading to abdominal distension, increased gastric residuals, and intestinal paralysis. Postoperative solitary human mild diet may be protective in neonates with a single ventricle type of CHD with improved short-term growth and decreased risk of NEC after cardiac surgery (29).

Although cardiogenic NEC is thought to be associated with mesenteric hypoperfusion and hypoxia in the gut, which may be attributed to low cardiac output syndrome, the impact of a concomitant reduction in cerebral perfusion causing poorer *ND outcome* after 1 year remains open. Cerebral hypoperfusion during the acute postoperative phase coupled with negative effects on the microbiota may lead to impaired ND outcome. Both pathophysiological mechanisms have been demonstrated to contribute to poorer ND outcome in preterm newborns. For extremely preterm infants, NEC has been described as a significant risk factor for impaired ND outcome. In these patients, gut microbiota seems to be a relevant factor for the development of the gut, immune system, and brain, described as gut–brain axis linking the gut microbiota and ND outcome (30).

Prolonged hospital stay after cardiac surgery is associated with worse ND outcome at 6 years of age (31) and serves as a surrogate marker for medical complexity. Therefore, NEC contributes as one important risk factor besides others determining a more severe postoperative clinical course leading to longer ICU stay and total hospital stay due to a higher postoperative morbidity. Nevertheless, the impact of surgery determined by the length of CPB time, cross-clamping time, and the need for postoperative ECMO did not influence the risk for postoperative NEC in our cohort (Table 1).

In the future, the management and prevention of postoperative NEC may include clear diagnostic and therapeutic strategies including balanced nutrition regimens and systematic register-based research.

Despite the prospective design of this multicentric observational registry, some *limitations* have to be taken into account. This includes that the perioperative management of the involved centers is not completely standardized. Furthermore, it would be very interesting to include more details regarding the perioperative management of nutrition, i.e., type, frequency, and amount of nutrition, but this is not part of the ORCHID registry and could not be standardized due to the multicentric study design. We only evaluated postoperative NEC due to the availability in the Swiss ORCHID, since preoperative NEC data are not collected. Furthermore, cerebral MRI was not analyzed systematically. The ND outcome was so far only evaluated early at 1 year of age and does not represent the longer-term outcome at school age.

## Conclusions

Postoperative cardiogenic NEC is associated with longer ICU and hospital length of stay and contributes together with other complications to impaired ND outcome at 1 year of age. In the future, Swiss ORCHID may offer a nationwide research platform to better understand the impact of perioperative risk factors on the long-term ND outcome.

## Data Availability

The datasets presented in this article are not readily available due to the ethical regulations (Basec-2022-00689). Requests to access these datasets may be directed to walter.knirsch@kispi.uzh.ch.

## The current composition of the Swiss ORCHID group

Lausanne: Nicole Sekarski, Julia C. Natterer, Juliane Schneider, Christelle L`Ebraly, Rene Pretre, Amir-Reza Hosseinpour. Geneva: Maya S. Bouhabib, Angelo Polito, Cristina Borradori-Tolsa, Tornike Sologashvili. Berne: Damian Hutter, Lena Kaiser, Marc Raphael Pfluger, Martin Glöckler, Katharina Fuhrer-Kradolfer, Sebastian Grunt, Therese Fahrni, Alexander Kadner. Zürich: Walter Knirsch, Michael von Rhein, Ruth Etter, Verena Rathke, Janet F. Kelly-Geyer, Beatrice Latal, Hitendu Dave, Robert Cesnjevar. Basel: Mark Brotzmann, Aarau: Hannah Kümin, Rachel Kusche, Chur: Christa Killer, St.Gallen: Ursula Speckle, Winterthur: Regula Schmid, Bellinzona: Barbara Goeggel-Simonetti, Solothurn: Letizia von Laer, Münsterlingen: Seraina Calonder Faas, Biel: Margreet Duetz, Neuchatel: Marc Ecoffey, Fribourg: Marie Pascale Metrailler, Baden: David Wille, Luzern: Berenice Bubl.

## Further collaborators in the period of the data used

Aarau: Andrea Capone, Luzern: Florian Bauder, Winterthur: Ulla Jochumsen, Biel: Lena-Marie Gerecke. Technical responsibility for the database and hosting: Mark Adams (Swiss Neonet).
